# A refined set of RxNorm drug names for enhancing unstructured data analysis in drug safety surveillance

**DOI:** 10.3389/ebm.2025.10374

**Published:** 2025-05-02

**Authors:** Wenjing Guo, Fan Dong, Jie Liu, Aasma Aslam, Tucker A. Patterson, Huixiao Hong

**Affiliations:** National Center for Toxicological Research, U.S. Food and Drug Administration, Jefferson, AR, United States

**Keywords:** adverse drug events, pharmacovigilance, natural language processing, database, DrugBank

## Abstract

Adverse drug events are harms associated with drug use, whether the drug is used correctly or incorrectly. Identifying adverse drug events is vital in pharmacovigilance to safeguard public health. Drug safety surveillance can be performed using unstructured data. A comprehensive and accurate list of drug names is essential for effective identification of adverse drug events. While there are numerous sources for drug names, RxNorm is widely recognized as a leading resource. However, its effectiveness for unstructured data analysis in drug safety surveillance has not been thoroughly assessed. To address this, we evaluated the drug names in RxNorm for their suitability in unstructured data analysis and developed a refined set of drug names. Initially, we removed duplicates, the names exceeding 199 characters, and those that only describe administrative details. Drug names with four or fewer characters were analyzed using 18,000 drug-related PubMed abstracts to remove names which rarely appear in unstructured data. The remaining names, which ranged from five to 199 characters, were further refined to exclude those that could lead to inaccurate drug counts in unstructured data analysis. We compared the efficiency and accuracy of the refined set with the original RxNorm set by testing both on the 18,000 drug-related PubMed abstracts. The results showed a decrease in both computational cost and the number of false drug names identified. Further analysis of the removed names revealed that most originated from only one of the 14 sources. Our findings suggest that the refined set can enhance drug identification in unstructured data analysis, thereby improving pharmacovigilance.

## Impact statement

Adverse drug events are a significant concern for public health, necessitating accurate detection in drug safety surveillance. While unstructured data is a valuable source for identifying adverse drug events, effective analysis depends on a comprehensive and accurate list of drug names. Although RxNorm is recognized for providing standardized drug names, its effectiveness in unstructured data analysis remains unassessed. Our research refined the list of RxNorm drug names to improve its suitability for unstructured data analysis. By removing duplicates, excessively long names, false names, and replaceable names, we created a more accurate and efficient list of drug names. Testing this refined set on drug-related PubMed abstracts revealed improved accuracy and reduced computational costs compared to the original RxNorm list. This refined list of drug names enables more accurate monitoring of adverse drug events, providing a valuable tool for improving drug safety surveillance and protecting public health.

## Introduction

Adverse drug events (ADEs) are harmful responses to medications that pose significant risks to patients with millions of deaths and hospitalization annually [1]. Effective monitoring of ADEs through drug safety surveillance is crucial for protecting public health. Drug safety surveillance begins in clinical trials, where new drugs are rigorously tested for safety and efficacy. However, clinical trials are limited by short exposure periods and the size and diversity of the tested population [2]. Therefore, post-market drug safety surveillance is crucial to identify potential ADEs in a large population, particularly for drugs repurposed to treat COVID-19. For example, originally developed for the treatment of hepatitis C, Remdesivir was later evaluated for antiviral activity against other viruses and, in 2020, received FDA approval for the treatment of COVID-19. Traditionally, post-market surveillance relies on spontaneous adverse event reporting systems [3, 4]. In the United States, the Food and Drug Administration’s Adverse Event Reporting System (FAERS) [5] collects adverse event reports, medication error reports, and product quality complaints from various sources, including the MedWatch program. FAERS has been widely used to investigate drug safety issues [6–9]. However, FAERS relies on voluntary reporting, which can result in underreporting and delays in identifying ADEs. In recent years, unstructured text data has become valuable sources for investigating ADEs.

To effectively analyze unstructured data for drug safety surveillance, it is important to identify drugs and associated ADEs. One challenge for identifying drugs in unstructured data is different names used for the same drugs. The active ingredient, generic names, trade names, brand names, and even street names can be used to indicate the same drug in unstructured text. Using acetaminophen, a commonly used analgesic, as an example, Tylenol, Paracetamol, Panadol, Anacin, Feverall, Mapap, Ofirmev, Tempra, and APAP (the abbreviation for its chemical name, N-acetyl-para-aminophenol) are names used for the same drug in unstructured documents. The use of various names for the same drugs in unstructured data complicates accurate identification of drugs, making the standardization and normalization of drug names essential.

Various methods have been used in the standardization and normalization of drug names, including dictionary-based methods [10], rule-based systems [11–16], advanced machine learning models [17–20], and hybrid approaches [19]. Dictionary-based methods use comprehensive drug dictionaries built from various sources to identify drug names [10]. In these methods, a comprehensive dictionary like RxNorm is essential to ensure accurate recognition of complex or less common drug names [21].

Rule-based systems, on the other hand, rely on predefined patterns or contextual rules to identify drug names. These rules can be either composition-based, focusing on systematic naming conventions, or context-based, extracting names based on surrounding text features [22, 23]. Despite the rigidity and extensive manual effort required to develop and maintain these rules and dictionaries—especially given the evolving nature of language and the introduction of new terminology—both dictionary and rule-based methods remain crucial for establishing a baseline of accurate drug identification.

To enhance the matching and normalization processes, similarity algorithms such as Levenshtein distance [24], cosine similarity [25], and Jaccard index [25] can be used. These techniques measure the similarity between drug names and help link various names of the same drug to a standard drug name [26, 27], further improving the accuracy of drug name standardization.

With the increasing availability of annotated datasets, machine learning-based models have gained significant popularity in this field [10, 17–20, 28]. Notable techniques such as Conditional Random Forest (CRF) [29], Hidden Markov Models (HMM), Recurrent Neural Networks (RNN) [30], and Bi-directional Long Short-Term Memory CRF (BI-LSTM-CRF) [31–33], and Bidirectional Encoder Representations from Transformers (BERT) [15] have been employed for drug name identification and normalization. These models leverage various features, including domain-specific attributes and word representation features, to improve accuracy.

Hybrid approaches have also emerged, integrating multiple methods to capitalize on the strengths of different models while mitigating their weaknesses [19]. For example, a semi-supervised machine learning technique known as feature coupling generalization was applied to refine a drug name dictionary, which was constructed from sources such as DrugBank and PubMed, to enhance drug name recognition in unstructured textual data [19].

To create a drug name dictionary, different names for the same drug are linked to a standardized name. A comprehensive dictionary is essential for accurate drug identification and normalization. RxNorm [34], a standardized vocabulary developed by the National Library of Medicine (NLM), plays a key role in these processes. RxNorm compiles drug names from 13 different sources and further standardizes them under its own unique terminology, RxNorm, bringing the total to 14 distinct sources, enabling consistent linkage of various drug names across different databases. The integration of RxNorm with both rule-based and machine learning approaches enhances the identification and normalization of drug names.

Although RxNorm is widely used in clinical settings, such as electronic health records and clinical decision support systems [35], it faces several limitations when analyzing unstructured data. One significant issue is the extensive variability in the length of drug names within RxNorm, which can range from one to over 2000 characters. These extremely short or long names are seldom found in unstructured text. Moreover, RxNorm includes distinct entries, various drug formats, and dosages, which are typically omitted when discussing experience with drugs in unstructured text. Even when such details are mentioned, they are often inconsistent and incomplete.

Additionally, RxNorm’s approach of combining drug names with specific dosages as separate entries can lead to multiple hits for the same drug in a single text. For example, “Acetaminophen” and “Acetaminophen 325 mg” are distinct entries in RxNorm. If both terms are included in a drug name dictionary, a sentence like “Acetaminophen 325 mg caused my mom’s liver injury” could lead to two matches—one for “Acetaminophen” and another for “Acetaminophen 325 mg”— resulting in redundant counts of the adverse event. These complexities stress the need for a refined set of drug names to improve the accuracy and efficiency of drug identification in unstructured data.

The purpose of this study is to develop an enhanced set of drug names from RxNorm, specifically tailored for identifying drug names in unstructured data for drug safety surveillance. By refining the existing drug names in RxNorm, this study aims to address current limitations and improve the accuracy and efficiency of drug identification in unstructured data.

## Materials and methods

### Study design

**FIGURE 1:**
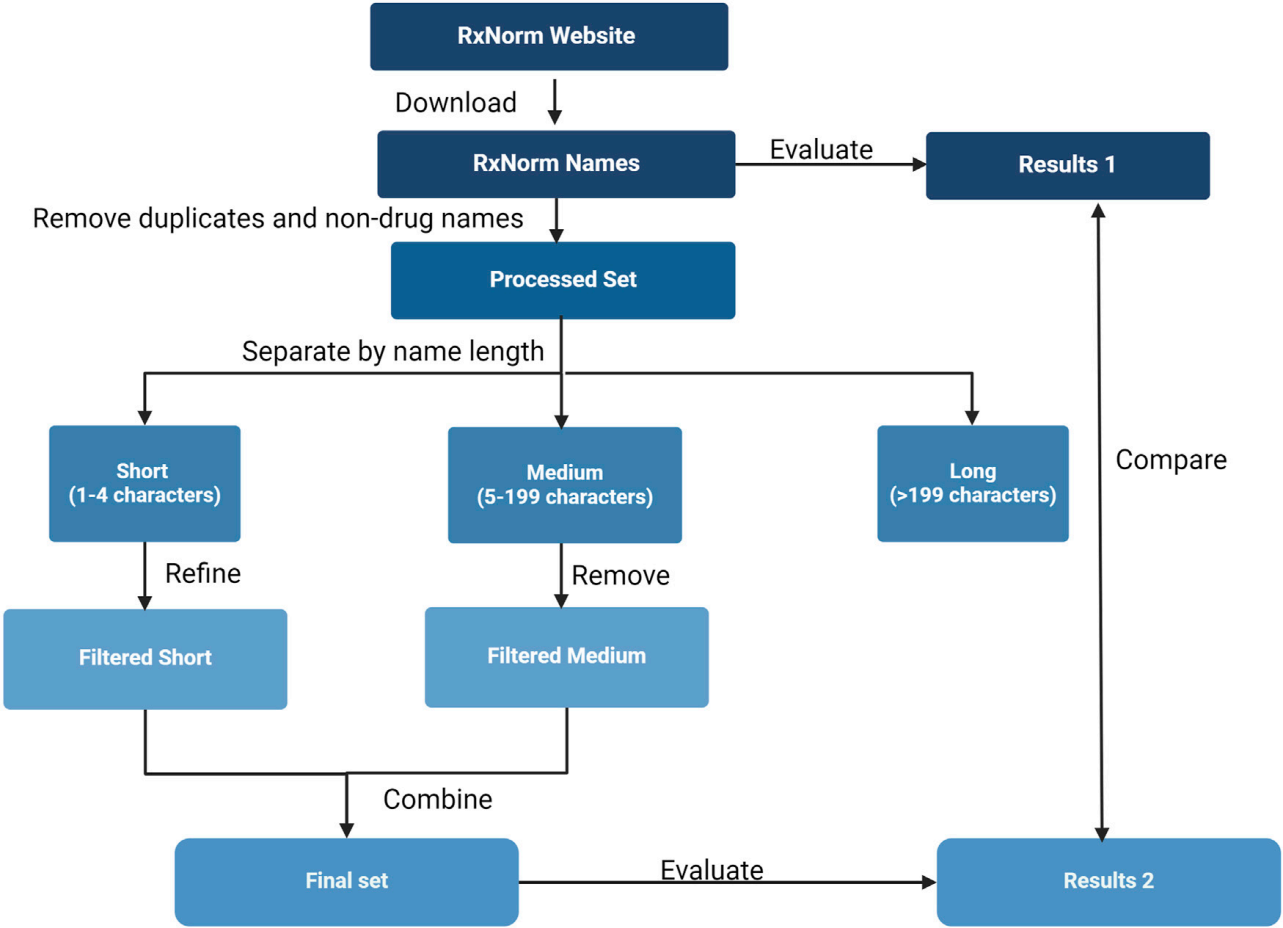
Study overview. The flowchart illustrates the procedures used to generate and evaluate a refined set of drug names from RxNorm, including extraction of drug names from the RxNorm website, removal of duplicates, filtering false names, discarding names that likely lead to redundant occurrence counts in unstructured data analysis, and evaluating accuracy and efficiency of the refined set.

The workflow for generating this refined set and assessing its accuracy and efficiency is depicted in Figure 1. Initially, a comprehensive list of drug names was downloaded from the RxNorm database. This was followed by a systematic process of removing duplicates, incorrect names, and names that could potentially cause inaccurate counts in unstructured data analysis. Drug names were classified into three categories and filtered out by those with fewer than 4 characters, those with between 5 and 199 characters, and those with 200 or more characters.

### Data sources

RxNorm file released on July 3, 2023 (RxNorm_full_07032023.zip) was downloaded from RxNorm repository [36]. The “RXNCONSO.RRF” file within this package was used to extract drug names. Specifically, drug names were obtained from the “STR” (string) column, while their corresponding types were identified from the “TTY” (type of terms) column, which includes categories such as brand name, synonyms, and others.

To ensure relevance, name types not associated with specific drugs were excluded based on the guidelines provided in the RxNorm technical documentation [37]. For instance, terms like dose form, dose form group, and special category—which describe routes of administration rather than specific drugs—were removed. The source of each drug name is indicated in the “SAB” (source abbreviation) column: ATC (Anatomical Therapeutic Chemical Classification System), CVX (Vaccines Administered), DB (DrugBank), GS (Gold Standard Drug Database), MMSL (Micromedex RED BOOK), MMX (Micromedex), MSH (Medical Subject Headings), MTHCMS (CMS Formulary Reference File), MTHSPL (FDA Structured Product Labeling), NDDF (First Databank), RXNORM (RxNorm itself), SNOMED (SNOMED Clinical Terms), USP (United States Pharmacopeia), and VANDF (Veterans Health Administration National Drug File).

To evaluate the extracted drug names, a dataset of 18,000 drug-related PubMed abstracts was prepared. These abstracts were retrieved by searching PubMed using the keyword “drug” via the Entrez Programming Utilities [38] (E-Utlilities) developed by the National Center for Biotechnology Information (NCBI). To comply with NCBI guidelines, we designated an email address for Entrez queries. On 22 May 2024, we generated a search query using the keyword “drug” without imposing any timeframe restrictions, ensuring the retrieval of all available abstracts up to that date. Entrez was used to retrieve 20,000 PubMed abstract IDs matching this query. Due to the limitation on the number of abstracts that can be fetched in a single request, we retrieved the IDs in two batches, with each batch containing 10,000 IDs. Abstracts were fetched and output for each batch. Although 20,000 IDs were obtained, 18,520 abstracts were successfully retrieved due to some missing entries. Ultimately, we used the first 18,000 abstracts, choosing this round number to simplify subsequent calculations.

### Refinement of RxNorm drug names

The first step is to remove duplicates and exclude drug names that are not associated with specific drugs. This includes eliminating terms that describe dose form, dose form group, and special category—such as “oral tablet,” “chewable product,” and “medical supplies”—since these are not linked to particular drugs and should, therefore, be excluded. Brand and generic drug names were retained to ensure comprehensive drug identification. For example, both Daytrana (patch) and Ritalin (oral tablet) were included as brand names for methylphenidate. This approach ensures that drug identification focuses on the medication itself while preventing redundant counts based on formulation differences. However, we recognize that ADEs can sometimes be associated with the delivery method rather than the active ingredient. For instance, systemic methylphenidate may be linked to behavioral effects like aggression, while transdermal formulations such as Daytrana may cause localized reactions like rash.

For drug names with four or fewer characters such as APAP (Acetaminophen), ASA (Aspirin), and HCTZ (Hydrochlorothiazide), their use frequency in unstructured data were tested in 18,000 drug-related PubMed abstracts to remove those that would rarely appear in drug-related documents. Drug names that were not found in these abstracts were considered rare and removed. We used the “en_core_web_sm” model from the spaCy [39] natural language processing (NLP) library to identify and count occurrences of these drug names within the abstracts. Each abstract was tokenized, and both tokens and drug names were converted to lowercase for consistency. We then compared each token against the list of drug names, recording an occurrence whenever a match was found. Drug names with zero occurrences were excluded from the final list.

For drug names with five to 199 characters, we examined their potential redundant occurrences in unstructured data analysis. If a drug name contains another drug name, leading to redundant counts, it was discarded. To identify distinct drug names that overlap with discarded names but not with other distinct names, we split each drug name into words using the Python’s “re.split” function (version 3.11.7 in Anaconda). The names were then sorted by word count. We checked if the words in a drug name contained all words of another name. If a drug name that does contain all the words of any other names, it was removed. Drug names with 199 or more characters were removed entirely, as they are unlikely to appear in real-world unstructured texts.

### Assessment of the refined set

To evaluate the efficiency and accuracy of the refined set of drug names in unstructured data analysis, we conducted drug identification on the 18,000 drug-related PubMed abstracts. The refined and original drug names were converted to lowercase and tokenized using the “en_core_web_sm” in spaCy. These tokenized drug names were used to create matching patterns, which were added to spaCy’s PhraseMatcher. Each abstract was tokenized, and the PhraseMatcher compared each sequence of tokens against the created matching patterns. When a match was found, the drug name was recorded.

Efficiency was measured by comparing the computational time required for both the refined and original RxNorm drug name sets. Accuracy was calculated as the ratio of drug names identified within the abstracts to the total number of drug names, for both the refined and original sets.

## Results

### Refinement of drug names

Table 1 provides a summary of the percentages of words removed at each stage of the refinement process, offering a clearer overview of the impact of our filtering criteria.

**TABLE 1:** Summary of removed words for each drug name type.

Name type	Percentage of removed words
Duplicates	23.61
Non-drug Names	0.09
Drug Names with less than 5 characters	0.06
Drug Names with 5-199 characters	65.78
Drug Names with >200 characters	1.53

### Download and processing of drug names

To refine the drug names in RxNorm, we downloaded the RxNorm file released on July 3, 2023, from the RxNorm website [40]. The “RXNCONSO” file in the downloaded zipped files was used to obtain drug names and other related information, with drug names stored in the “STR” column. A total of 1,143,201 drug names were retrieved from which 269,931 duplicates were identified and removed. Then, we examined the types of the retained drug names to remove those not containing specific drug information. According to the RxNorm technical documentation [41], three term types (DF, DFG, SC) pertain to administrative details rather than specific drugs. We removed 1,009 drug names belonging to these categories.

### Drug names with four or fewer characters

We used 18,000 drug-related PubMed abstracts to evaluate the occurrence of drug names with four or fewer characters. Out of 1260 drug names, 687 had zero occurrences and were discarded. The occurrences of the remaining drug names with the abstracts are provided in Supplementary Table S1.

**FIGURE 2:**
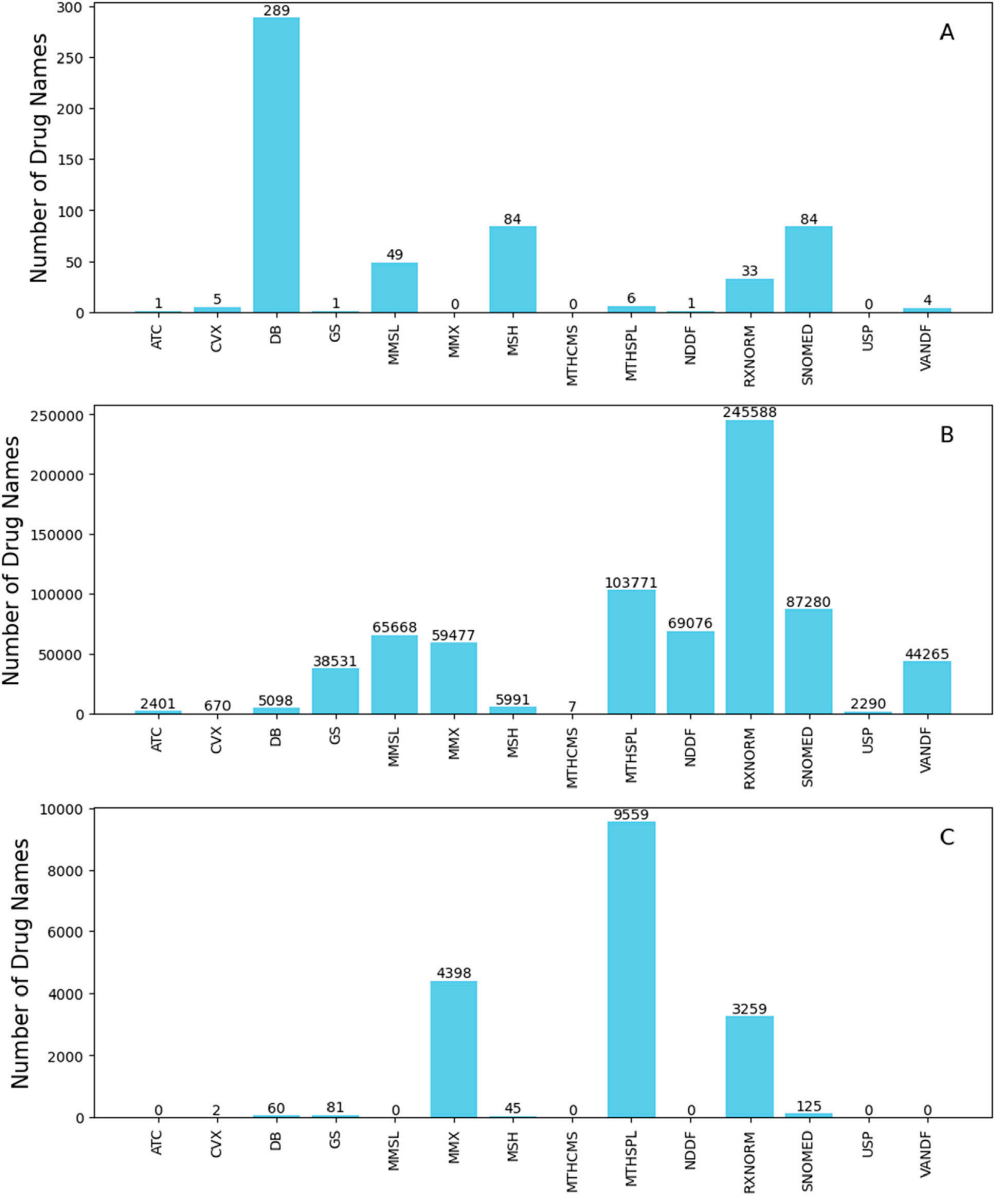
Source distribution of the removed drug names that only originate from a single source for names with four or fewer characters **(A)**, names with five to 199 characters **(B)**, and names with 200 or more characters **(C)**. The y-axes give number of names and x-axes depict name sources. Abbreviations: ATC (Anatomical Therapeutic Chemical Classification System), CVX (Vaccines Administered), DB (DrugBank), GS (Gold Standard Drug Database), MMSL (Micromedex RED BOOK), MMX (Micromedex), MSH (Medical Subject Headings), MTHCMS (CMS Formulary Reference File), MTHSPL (FDA Structured Product Labeling), NDDF (First Databank), RXNORM (RxNorm itself), SNOMED (SNOMED Clinical Terms), USP (United States Pharmacopeia), and VANDF (Veterans Health Administration National Drug File).

We further analyzed the sources of the 687 discarded names. Our analysis showed that the majority originated from a single source among the 14 in RxNorm, indicating that drug names from a single source are unlikely to appear in unstructured drug-related texts. This result is not surprising, as these names lack corroboration from other sources. We also examined the source distribution of these 557 names. As shown in Figure 2A, DrugBank had the highest number (289), followed by SNOMEDCT_US (84) and MSH (84). In total, DrugBank, SNOMEDCT_US, and MSH, contained 628, 250, and 233 drug names with four or fewer characters, respectively. This indicates that approximately 46%, 34%, and 36% of such names from DrugBank, SNOMEDCT_US, and MSH were excluded. In contrast, sources like NDDF and MTHSPL had fewer names of this length and a lower removal rate, with only 1 out of 60 from NDDF and 6 out of 62 from MTHSPL being removed.

### Drug names with five to 199 characters

For drug names with five to 199 characters, we excluded those that could lead to redundant occurrence counts in unstructured data analysis. For example, using both original drug names “Acetaminophen” and “Acetaminophen 325 MG Oral Tablet” to identify adverse events for drugs in the text “my brother had headache after take acetaminophen 325 MG tablet”, might lead to two counts for the adverse event “headache” when only one should be recorded. Therefore, drug names that contain other names were removed, while distinct names without overlaps were retained. Out of 853,472 names with five to 199 characters, 101,491 are distinct names and were retained, whereas 751,981 names, which contain other names, were removed.

A significant portion of the removed names (730,113 out of 751,981) originate from only one of the 14 sources in RxNorm. The source distribution of these removed single-sourced names is shown in Figure 2B. Most of these drug names came from RxNorm, followed by MTHSPL, SNOMEDCT_US, NDDF, and MSSL. Specifically, RxNorm, MTHSPL, SNOMEDCT_US, NDDF, and MMSL provided 279,465, 121,035, 108,421, 99,054, and 91,270 drug names with five to 199 characters, respectively. The removal rates for these names are notably high: 87.8% for RxNorm, 85.7% for MTHSPL, 80.4% for SNOMEDCT_US, 71.9% for MMSL, and 69.7% for NDDF. In contrast, only 16.4% (5,098 out of 31,041) of the names with five to 199 characters from DrugBank were removed.

### Drug names with 200 or more characters

Drug names with 200 or more characters are rarely used in unstructured data and, therefore, were excluded. A total of 17,529 such drug names were found in RxNorm and excluded. All these names originated from a single source, with the source distribution depicted in Figure 2C.

### Evaluation of the refined drug names set

**FIGURE 3:**
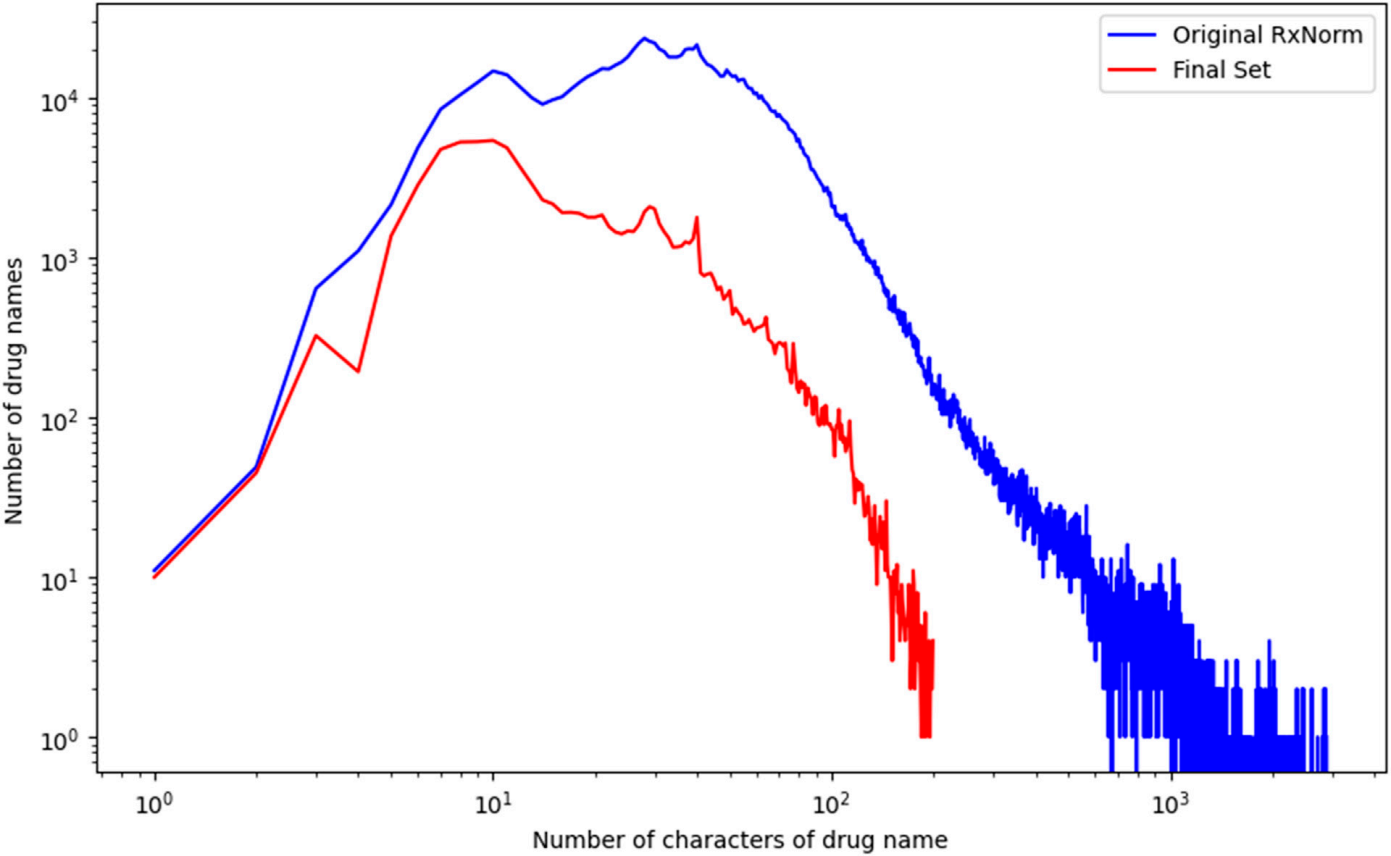
Comparison of name length between the refined set and the original RxNorm set. The y-axis shows the number of drug names, and the x-axis indicates name length. Name lengths were color coded in red for the refined sets and in blue for the original RxNorm set.

The refined set of drug names include 573 names with four or fewer characters and 101,491 names with five to 199 characters. We analyzed the distribution of drug name lengths between the refined set and the original RxNorm set. As shown in Figure 3, longer drug names were less likely to be retained in the refined set. This suggests that longer drug names are more prone to generating redundant occurrence counts in unstructured data analysis compared to shorter drug names and were thus discarded.

**FIGURE 4:**
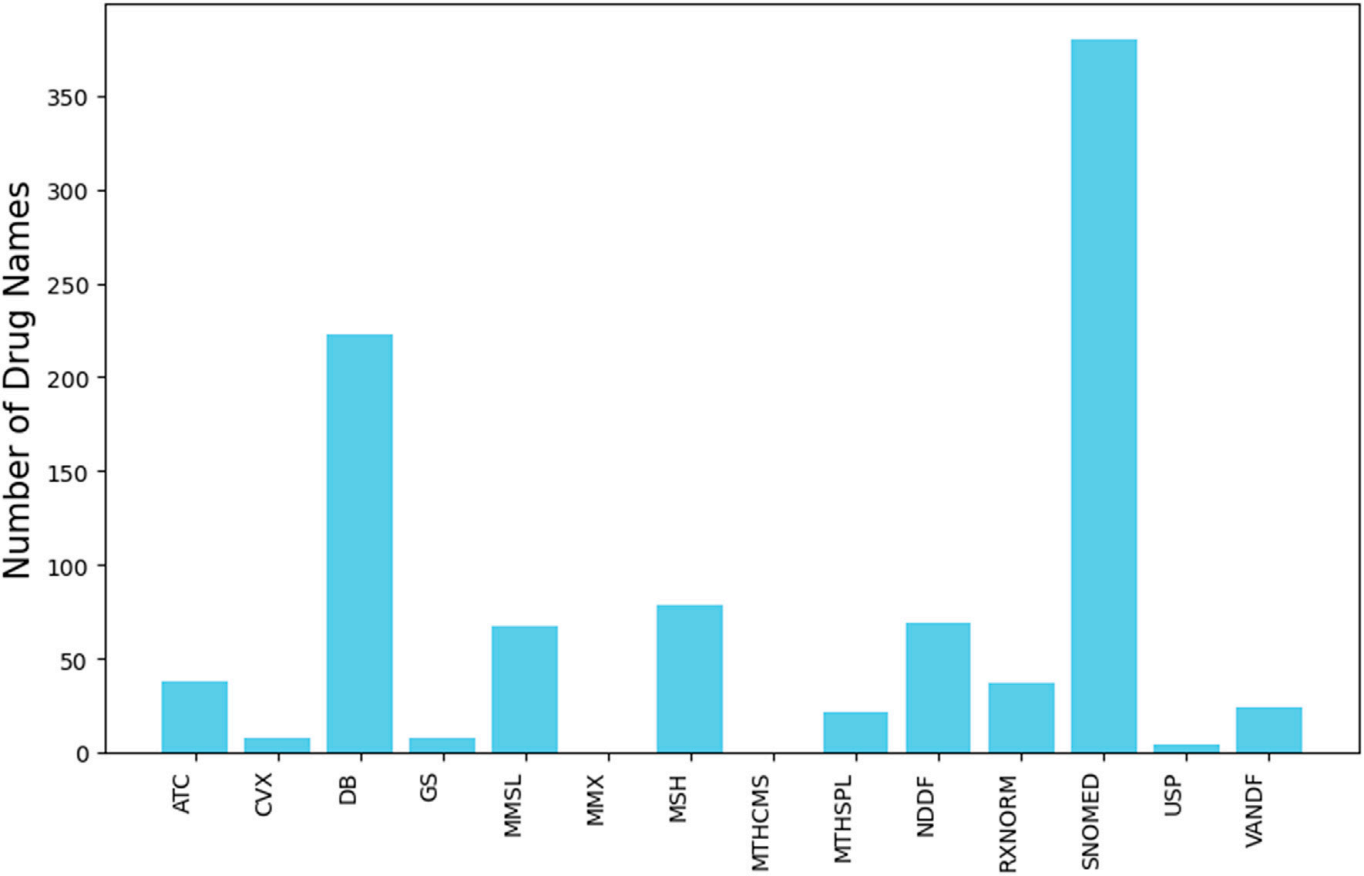
Source of original RxNorm drug names that were excluded from the refined set but identified in the PubMed abstracts. The y-axis represents number of drug names and the x-axis depicts sources. Abbreviations: ATC (Anatomical Therapeutic Chemical Classification System), CVX (Vaccines Administered), DB (DrugBank), GS (Gold Standard Drug Database), MMSL (Micromedex RED BOOK), MMX (Micromedex), MSH (Medical Subject Headings), MTHCMS (CMS Formulary Reference File), MTHSPL (FDA Structured Product Labeling), NDDF (First Databank), RXNORM (RxNorm itself), SNOMED (SNOMED Clinical Terms), USP (United States Pharmacopeia), and VANDF (Veterans Health Administration National Drug File).

To evaluate the efficiency and accuracy of the refined set of drug names, we used 18,000 drug-related PubMed abstracts. Our results revealed that 3,065 names were identified in the abstracts, with lengths ranging from 1 to 46 characters. When we evaluated the original RxNorm set using the same abstracts, we found 4,471 names with lengths ranging from 1 to 66 characters. The additional 1,046 names that RxNorm identified in the abstracts were either false drug names or names likely leading to redundant occurrence counts in unstructured data analysis. These names were excluded from the refined set, with the majority originating from DrugBank and SNOMEDCT_US, as shown in Figure 4. Our results reveal that the refined set of drug names improved drug identification accuracy in analyzing unstructured texts compared to the original RxNorm set.

The efficiency of the refined set of drug names was measured using the computational time required to analyze the abstracts. The analysis using the refined set took 1,910 s, while using the original RxNorm set took 6,301 seconds—over three times longer. Our results demonstrate a significant improvement in efficiency when analyzing unstructured data, making the refined set more suitable for real-time drug safety surveillance.

## Discussion

Artificial intelligence is increasingly playing a critical role in evaluating drug safety and chemical toxicity. By harnessing machine learning algorithms and computational models, artificial intelligence can predict adverse effects, identify toxic compounds, and improve pharmacovigilance efforts. There are two main types of data involved: structured and unstructured. Due to their distinct formats and organization, machine learning techniques are applied differently to each. Structured data is well-organized and easily interpretable by machines, making it a natural fit for a wide range of safety assessments and toxicity endpoints [40–53]. In contrast, unstructured data lacks a predefined format, which makes it more challenging to process and analyze. To effectively apply machine learning techniques, such as natural language processing and recurrent neural networks, to unstructured data in pharmacovigilance, a reliable and comprehensive set of drug names is essential.

In this study, we generated a refined set of drug names from RxNorm to improve the accuracy and efficiency of drug identification in unstructured data. The original RxNorm set contained duplicates, non-specific drug names, and names that were either too long or too short, which hindered effective drug identification in unstructured data. Our objective was to exclude such names from analysis of unstructured texts. The refined set was evaluated using 18,000 drug-related PubMed abstracts, demonstrating enhanced accuracy and efficiency in drug identification, thereby potentially improving drug safety surveillance through unstructured data analysis.

Single-sourced drug names, originated from only one of the 14 sources in RxNorm, are generally less reliable than names corroborated by multiple sources. These single-sourced names tend to cause incorrect identification or generate redundant occurrence counts when analyzing unstructured data, affecting both the accuracy and efficiency of drug identification. Our results revealed that the majority of the removed names were single-sourced, highlighting the importance of utilizing drug names validated by multiple sources.

Furthermore, most of the removed single-sourced names originated from FDA Structured Label, RxNorm, and SNOMEDCT_US. These sources serve distinct roles in drug information management. The FDA Structured Product Label provides comprehensive regulatory drug details, including dosage, formulation, and safety information, to ensure clarity and reduce medication errors. RxNorm standardizes drug names by linking ingredients, strengths, and dosage forms, facilitating interoperability across electronic health systems. SNOMED CT, on the other hand, is primarily used for clinical documentation and coding within electronic health records.

RxNorm integrates drug names from multiple external sources; however, not all names from contributing databases are necessarily included. Furthermore, many drug names appear in multiple sources within RxNorm, potentially leading to redundant listings. To mitigate this, our analysis systematically identified and removed duplicate drug names contributed by multiple sources, ensuring that each unique drug name was counted only once. While these structured resources are essential for clinical and regulatory use, their detailed naming conventions can complicate drug identification in unstructured data. Refining these names is crucial to enhance their applicability in text-based analyses.

On the other hand, sources like DrugBank and MSH showed varying levels of reliability across different lengths of drug names. For drug names with four or fewer characters, DrugBank had a relatively high removal rate of 46%, indicating that many of these names are unlikely to appear in unstructured data. However, the removal rate for DrugBank drug names with five to 199 characters significantly reduced to 16.4%, suggesting that these names are more reliable in unstructured data analysis. Similarly, MSH had a high removal rate of 36% for names with four or fewer characters and a lower rate of 24% for names with five to 199 characters. Our results suggest that more caution is needed when using short names from DrugBank and MSH in unstructured data analysis for drug safety surveillance compared to their longer names.

Despite the improvements in accuracy and efficiency demonstrated by the refined set, some limitations should be noted. First, our refined set of drug names is not error-free for unstructured data analysis, and some unsuitable names may persist. For example, short drug names in the refined set might include common words that, depending on the context, do not refer to drugs. Second, as RxNorm is primarily composed of professionally used names, it may not capture the variations found in street names or slang used in non-professional documents. Third, because RxNorm is updated monthly, regular updates are necessary to maintain the accuracy and relevance of the refined set. Finally, our evaluation was limited to 18,000 drug-related PubMed abstracts. Although we focused on abstracts containing the keyword “drug” to increase the likelihood of identifying drug names, these abstracts may not represent other unstructured real-world data. We selected the keyword “drug” to maximize the inclusion of abstracts that explicitly mention specific drug names. Alternative terms such as “medications” or “pharmacologic” were not used, as they are often associated with broader discussions on treatment strategies, pharmacological mechanisms, or drug classes rather than individual drug names. Additionally, a composite search incorporating all relevant MeSH terms was not conducted to ensure consistency with prior studies that employed keyword-based retrieval for drug-related text analysis. This approach maintains methodological alignment while optimizing the extraction of relevant drug name mentions.

Further efforts are needed to enhance the refined set. One such effort involves evaluating the set more comprehensively using diverse unstructured data. Additionally, the refined set could be improved by integrating advanced algorithms and machine learning techniques. Machine learning algorithms, particularly those involving similarity measurements, could be trained to recognize and link synonymous drug names, thereby improving accuracy. Natural language processing techniques like BERT could also be employed to better understand the context in which drug names appear, further enhancing accuracy. Finally, developing automated processes for updating the drug names in the dataset is crucial. As RxNorm updates its dataset monthly, maintaining the refined set through an automated update process will ensure its continued reliability for unstructured data mining in drug safety surveillance.

## Conclusion

The development of the refined set of drug names from RxNorm has shown significant improvements in the accuracy and efficiency of drug identification in unstructured data. This refined dataset could be valuable for extracting drug-related information from unstructured data, thereby supporting more effective monitoring and management of drug safety through unstructured data analysis. Our study also highlights the importance of addressing the limitations of existing drug names when used for unstructured data mining, particularly in the context of drug safety surveillance.

## Data Availability

The raw data supporting the conclusions of this article will be made available by the authors, without undue reservation.
